# Dynamic Roles of RNA and RNA Epigenetics in HTLV-1 Biology

**DOI:** 10.3390/v17010124

**Published:** 2025-01-17

**Authors:** Emily M. King, Amanda R. Panfil

**Affiliations:** 1 Center for Retrovirus Research, Department of Veterinary Biosciences, The Ohio State University, Columbus, OH 43210, USA; 2 Center for RNA Biology, Comprehensive Cancer Center, Department of Veterinary Biosciences, The Ohio State University, Columbus, OH 43210, USA

**Keywords:** ATLL, epigenetic, HTLV-1, m^6^A, RNA

## Abstract

Since the discovery of RNA in the early 1900s, scientific understanding of RNA form and function has evolved beyond protein coding. Viruses, particularly retroviruses like human T-cell leukemia virus type 1 (HTLV-1), rely heavily on RNA and RNA post-transcriptional modifications to regulate the viral lifecycle, pathogenesis, and evasion of host immune responses. With the emergence of new sequencing technologies in the last decade, our ability to dissect the intricacies of RNA has flourished. The ability to study RNA epigenetic modifications and splice variants has become more feasible with the recent development of third-generation sequencing technologies, such as Oxford nanopore sequencing. This review will highlight the dynamic roles of known RNA and post-transcriptional RNA epigenetic modifications within HTLV-1 biology, including viral *hbz*, long noncoding RNAs, microRNAs (miRNAs), transfer RNAs (tRNAs), R-loops, N6-methyladenosine (m^6^A) modifications, and RNA-based therapeutics and vaccines.

## 1. Introduction

Human T-cell leukemia virus type 1 (HTLV-1) is an oncogenic retrovirus and the causative agent of several human diseases, including adult T-cell leukemia/lymphoma (ATLL), HTLV-1-associated myelopathy/tropical spastic paraparesis (HAM/TSP), and several inflammatory conditions (uveitis, keratitis, conjunctivitis, and dermatitis) [1,2,3]. An estimated 5–10 million people are infected worldwide, with pockets of endemic infection in Japan, Africa, the Caribbean islands, and South America. HTLV-1-associated diseases develop in ~5–10% of carriers following a prolonged clinical latency period of several decades [2,4,5]. However, current epidemiologic estimates likely underrepresent true infection rates given the modes of transmission (mother-to-child through breastfeeding, sexual contact, exposure to infected blood products), lack of large epidemiologic studies, and insufficient screening/prevention in many countries [6]. Infected individuals carry a 5% lifetime risk of developing ATLL; however, this risk increases to 25% for those infected at birth [1,7,8]. This increased risk is mainly attributed to the high HTLV-1 proviral load in breastmilk from infected mothers, a predictor and correlate of disease development [5]. Risk of disease development can be further exacerbated by prolonged (at least >6 months) durations of breastfeeding [5]. The high incidence of neoplasia makes HTLV-1 one of the most oncogenic pathogens to date.

HTLV-1 contains a small positive-sense RNA genome that is approximately 9 kb in size [9]. Two copies of viral RNA are packaged into the viral particle [10]. The viral genome encodes structural and enzymatic genes present in all retroviruses (Gag, Pro, Pol, and Env), as well as regulatory and accessory genes from a unique ‘pX’ region located 3′ to Env [11]. The proviral genome is flanked by 5′ and 3′ long terminal repeats (LTR) that are generated during the reverse transcription process [12]. Of the HTLV-1 gene products, Tax and Hbz have been strongly implicated in T-cell transformation and oncogenesis [13,14]. Early proviral transcription and cell immortalization are driven by Tax protein through its interaction with several important signaling pathways involved in cell survival, proliferation, and genomic stability [15,16]. Tax and the cAMP response element-binding protein (CREB) form a complex that is then recruited to a conserved set of 21-base pair repeats in the 5′ U3 region known as Tax responsive elements (TREs) [17]. CREB then recruits cofactors p300 and CBP to the viral 5′ LTR promoter, driving the transcription of sense-genome-strand-encoded gene products [18]. Tax also influences several cell signaling pathways, including persistent activation of NF-κB [19,20]. Tax is oncogenic, but if left unregulated, the infected cell typically undergoes apoptosis or immune recognition [21,22,23,24]. Therefore, tight control of viral gene expression is paramount for infected cell survival. Tax expression is typically low or undetectable in most ATL cells [25]. However, recent evidence has revealed that Tax is expressed in a minor fraction of leukemic cells at any given time, and this expression is spontaneously switched between ‘on’ and ‘off’ states [26]. This transient expression of Tax induces NF-κB expression to promote survival and is critical for maintaining the infected cell population [26].

Encoded from the antisense strand of the proviral genome, Hbz can also inhibit Tax-mediated activation of viral transcription within the nucleus of infected cells [27]. By interacting with transcription factors, such as ATF-1, CREB, JunB, JunD, and/or c-Jun, through its basic leucine zipper domain, Hbz can sequester these factors away from Tax and inhibit Tax-mediated transcription [28]. Hbz can antagonize Tax activation of the classical NF-κB pathway by complexing with p65/RelA, preventing p65 translocation to the nucleus [29,30]. Beyond antagonizing the functions of Tax, Hbz also directly promotes proliferation of T cells in both its protein and mRNA forms. Hbz induces JunD expression to facilitate transcriptional activation, as well as impede the tumor suppressor function of ATF3 by preventing binding to p53 [31,32]. This proliferative effect is further supported by *hbz* mRNA, which has roles distinct from those of the Hbz protein (discussed below) [33]. In brief, *hbz* mRNA upregulates E2F1, a critical regulator of cell cycle progression [33]. Taken together, Hbz is critical for establishment and maintenance of chronic viral infection, while Tax is essential for cellular immortalization/transformation. Importantly, the balance between Tax and Hbz is a central feature of the viral lifecycle and directly contributes to the pathogenic potential of HTLV-1.

RNA is critical for gene expression and can assume myriad forms with varied roles, including protein coding (mRNA), transcriptional regulation (lncRNA), splicing (snRNA), and translation (rRNA, tRNA, miRNA) [34,35,36,37,38]. The primary structure of RNA is composed of nucleosides attached by 5′-3′ phosphodiester bonds between ribose sugars [39]. Nucleoside bases consist of adenine, guanine, cytosine, and uracil, which are connected by hydrogen bonds. This base pairing by hydrogen bonding is responsible for the secondary structure of RNA [40]. RNA tertiary structure is conferred via RNA folding, creating a three-dimensional shape of helices and grooves [41]. In eukaryotes, RNA is transcribed from a DNA template using various RNA polymerases, including RNA polymerase I (rRNA, tRNA), RNA polymerase II (mRNA), and RNA polymerase III (tRNA, 5S rRNA) [42]. The newly synthesized RNA strand is nearly identical to the noncoding strand of DNA, apart from uracil being substituted in place of thymine. RNA transcription is terminated by various mechanisms based on the RNA polymerase responsible for driving transcription, including binding of the termination protein TTF-1 to a specific sequence of RNA (RNA polymerase I), use of a cleavage site and polyA tail (RNA polymerase II), and the less-understood usage of 3′ uracil bases for disengagement of the transcriptional complex (RNA polymerase III) [43,44,45].

Alterations in RNA processing have frequently been observed in both B- and T-cell leukemias, suggesting the essential role of RNA regulation in tumorigenesis [46,47,48,49,50,51,52,53]. Leukemic cell development has been associated with alterations in mRNA splicing, cleavage/polyadenylation, and nuclear export of transcripts. Dysregulated alternative splicing has emerged as a major mechanism of acute myeloid leukemia (AML) development, and often has significant prognostic consequences related to poor survival [54,55,56,57]. Alternative polyadenylation of mRNA has been observed in several leukemias and typically occurs within the 3′ untranslated region (UTR), changing the protein isoform [58]. mRNA export has been less studied in the context of leukemia, although one study found targeting the eIF4E-dependent mRNA export pathway correlated with improved clinical response in AML patients [59]. Noncoding RNAs (ncRNA) also play a significant role in leukemias [51,60]. One of the most common noncoding RNA networks in leukemogenesis is that of miRNA. miRNAs targeting tumor suppressor genes are downregulated, and those targeting oncogenic genes are upregulated, in B- and T-cell leukemias [61,62,63,64,65,66,67,68,69,70,71,72]. circRNAs have been shown to play a crucial role in both immunomodulation and disease development [73]. Various circRNAs have demonstrated activation of cell signaling pathways involving phosphoinositide 3-kinase (PI3K) and mitogen-activated protein kinase (MAPK), contributing to AML cell proliferation, survival, progression, and therapeutic resistance [73]. In addition, specific circRNAs (hsa_circ_0074995, _0074998, _0075001, _0075002, and _0075012) are overexpressed in AML cells, resulting in downregulation of Toll-like receptor signaling pathways, indicating a role for circRNAs in immune modulation [74]. Similarly, lncRNA profiles have been uncovered in both B- and T-cell leukemias, some of which have demonstrated significant roles in gene regulation [75]. The frequently studied lncRNA X-inactive specific transcript (Xist) plays a critical role in transcript silencing and often functions as a tumor suppressor in AML [76]. Overall, RNA plays a diverse role in the regulation of leukemic oncogenesis. This review will focus on the dynamic roles of known RNA and post-transcriptional RNA epigenetic modifications within HTLV-1 biology.

## 2. N-6 Methyladenosine (m^6^A)

N-6 methyladenosine (m^6^A) represents methylation at the N6 position of an adenosine base and is the most abundant post-transcriptional modification in eukaryotic RNA. This modification is often enriched near stop codons and 3′ UTRs, suggesting a critical role in cells [77,78,79]. Three groups of cellular proteins are responsible for the dynamic nature of m^6^A deposition, collectively termed writers, readers, and erasers. The writer proteins (methyltransferase-like 3 [METTL3] and methyltransferase-like 14 [METTL14]) are responsible for transferring a methyl group from an S-adenysyl methionine (SAM) molecule onto the sixth nitrogen of an adenosine base [80]. The methyl modification is typically deposited at DRACH motifs (where D = A, G, or U; R = purine; and H = A, C, or U) on nascent transcripts [78,81]. A small molecule inhibitor that targets METTL3 (STM2457) and therefore depletes m^6^A levels has been employed as an AML therapeutic [82,83]. Methyl modifications are removed by eraser proteins AlkB homolog 5 (ALKBH5) and fat-mass-and-obesity-associated protein (FTO). These demethylases catalyze the oxidative phosphorylation of m^6^A to regenerate an unmodified adenosine [84,85,86]. Transcripts modified by m^6^A are interpreted by ‘reader’ proteins (YTHDF1-3 and YTHDC1-2) [30,87,88,89,90,91], members of the YTH-domain-containing family of proteins, whose preference for m^6^A-modified RNA is specified by an aromatic cage that forms a hydrophobic pocket around the modified nucleotide [92]. Based on the body of research to date, YTHDF1 has been shown to promote CAP-dependent translation by interacting with the 5′ UTR-associated eIF3 protein [88]. Conversely, m^6^A modification in the 5′ UTR can promote CAP-independent translation by directly recruiting eIF3 [93]. Previous studies found that YTHDF2 regulates mRNA stability by recruiting the CCR4–NOT deadenylase complex to promote degradation of m^6^A-modified RNA [87,91]. The YTHDF3 reader protein has several reported overlapping functions with both YTHDF1 and YTHDF2, promoting both RNA translation and decay [90,94]. The functional roles of these cytoplasmic reader proteins remain somewhat controversial, likely due to their context-dependent functions. YTHDC1 is the only reader protein localized to the nucleus, where it promotes splicing and nuclear export of m^6^A-modified pre-mRNAs. YTHDC1 interacts with splicing factors SRSF3 and SRSF10, with SRSF3 further associating with nuclear RNA export factor 1 (NXF1) to facilitate RNA export [89]. The role of YTHDC2 has not yet been fully elucidated.

While m^6^A has been extensively documented in other retroviruses such as human immunodeficiency virus type 1 (HIV-1), it has only newly been qualified in the HTLV-1 lifecycle. Most recently, HTLV-1 viral RNA was found to be m^6^A-modified, with enrichment of m^6^A modification near the regulatory pX region of the viral genome [95]. Viral transcripts *tax* and *hbz* were also found to be m^6^A-modified in HTLV-1-transformed and ATLL-derived patient cell lines. Interestingly, global depletion of m^6^A using the inhibitor STM2457 had opposing effects on viral sense-derived *tax* (decreased) and antisense-derived *hbz* (increased) transcripts. Using overexpression and knockdown studies, it was found that YTHDF1 differentially regulates *tax* (inhibits) and *hbz* (promotes) expression, while YTHDC1 promotes viral transcript abundance in part through nuclear export of viral transcripts. This same study found that *tax* and *hbz* transcripts are bound by reader proteins YTHDF1 and YTHDC1 in T cells. Together, these results suggest that m^6^A modification of HTLV-1 RNA alters viral gene expression. Future studies are warranted to determine what aspects of viral transformation and/or disease development require YTH reader proteins.

## 3. R-Loops

R-loops consist of a three-stranded nucleic acid structure composed of an RNA–DNA hybrid and a displaced single-stranded DNA loop [96]. These naturally occurring structural elements regulate various cellular processes, including isotype switching, CRISPR-mediated genome editing, transcription-coupled nucleotide excision repair (TC-NER), chromatin structure, and transcription [97,98,99]. R-loop accumulation promotes DNA damage and subsequent genomic instability, which is known to occur in states of unregulated transcription and deficiencies in RNA splicing, processing, and export [100]. While the role of R-loops in the context of HTLV-1 infection is sparse, one study found that expression of Tax promotes R-loop accumulation, DNA damage, and senescence in an NF-κB-dependent manner [101]. This effect was largely mediated by TC-NER coupling factors (xeroderma pigmentosum F [XPF], xeroderma pigmentosum G [XPG], and Cockayne syndrome B [CSB]), which are detected in newly HTLV-1-infected cells but are deficient in ATLL-derived cells [101]. Thus, deficiencies in TC-NER and consequential R-loop accumulation may be a future source of therapeutic exploitation in ATLL patients.

## 4. Long Noncoding RNA (lncRNA)

lncRNAs are noncoding transcripts longer than 200 nucleotides that are derived from precursor mRNAs [102,103]. While many lncRNAs retain a 5′ cap and 3′ poly(A) tail, they differ from mRNA by containing fewer exons, weaker internal splicing signals, and alternative polyadenylation, thus resulting in nuclear retention [104,105,106,107]. lncRNAs are extensively involved in gene regulation by facilitating alternative assembly and function of nuclear bodies, controlling chromatin architecture, and facilitating transcriptional and post-transcriptional events [108,109,110,111,112,113,114]. lncRNAs have a negative charge, which, when coupled with the positively charged histone tails, promotes chromatin relaxation and facilitates DNA regulation [115]. lncRNAs can also regulate gene expression by inhibiting RNA polymerase II transcription factors, thus altering formation of genomic domains and assembling nuclear scaffolds/condensates such as paraspeckles, speckles, and perinuclear compartments [110,116,117,118,119]. Finally, lncRNAs can post-transcriptionally control gene expression by hijacking proteins implicated in mRNA turnover and scrounging regulatory RNAs through both sequestration and degradation [120,121,122,123].

The lncRNA ANRIL (antisense noncoding RNA in the INK4 locus) is elevated in both HTLV-1-infected cell lines and clinical ATLL samples [124]. Expression of ANRIL promotes cellular proliferation and represses apoptosis in vitro and in vivo by targeting EZH2 and the activation of the NF-κB pathway. ANRIL forms an ANRIL/EZH2/p65 ternary complex that further supports cellular proliferation through H3K27 trimethylation of the p21/CDKN1A promoter and consequential transcriptional silencing [124]. Given the documented role of ANRIL as a prognostic indicator in other leukemias, this lncRNA may serve as a potential future ATLL therapeutic target [125,126,127,128,129].

## 5. microRNA (miRNA)

Mature miRNA consists of a single-stranded RNA molecule that is 18–24 nucleotides long and is selected from an asymmetrical RNA duplex [130]. The mature miRNA is extensively implicated in RNA silencing through involvement in the RNAi pathway. However, in contrast to small interfering RNA (siRNA) or short hairpin RNA (shRNA) that are produced in plants or synthetically derived, miRNAs are the primary transcripts used naturally by the RNAi pathway in mammalian cells [131]. Unlike siRNA or shRNA, miRNAs are not fully complementary to their target RNA, resulting in translational repression and targeted degradation of transcripts rather than direct cleavage of RNA by the RNAi pathway [131].

Many miRNAs are synthesized by a canonical pathway, beginning with the synthesis of primary miRNA (pri-miRNA) in the nucleus by RNA polymerase II [132]. The nuclear enzyme Drosha in conjunction with DiGeorge syndrome critical region 8 (DGCR8) cleaves pri-miRNAs to produce precursor miRNA (pre-miRNA) [133]. Pre-miRNA is then exported to the cytoplasm by exportin-5 and processed by the Dicer complex with co-factors TAR RNA-binding protein (TRBP) and protein kinase R activator (PACT), resulting in double-stranded miRNAs [134,135,136,137,138]. Noncanonical generation of miRNA does not require Drosha or DGCR8, as these miRNAs are derived from other types of nuclear RNAs rather than being directly transcribed by RNA polymerase II [139,140]. Regardless of whether generated via the canonical or noncanonical pathways, after processing, the miRNA is loaded onto the Argonaute (Ago) protein. This results in the removal of one of the miRNA strands, resulting in a single-stranded guide RNA [141]. This guide RNA can then hybridize to the target RNA sequence, resulting in RNA silencing mediated by translational repression and degradation in processing bodies (P-bodies) [141]. It is estimated that miRNAs can regulate up to 60% of the total proteome [142].

### 5.1. Cell-Specific miRNA

The effects of miRNAs have been extensively documented in the context of HTLV-1 infection and are summarized in Table 1. While most miRNAs are directly associated with functions related to Tax or Hbz proteins, cellular miRNA has also been implicated in anti-viral activity. One of the first miRNAs implicated in the HTLV-1 lifecycle was cellular miR-28-3p, which inhibits HTLV-1 gene expression and replication by targeting a specific site within the genomic gag/pol viral RNA [143]. miR-28-3p is highly expressed in resting T cells, which are resistant to HTLV-1 infection [143,144,145]. This may be in part facilitated through induction of a post-entry block at the reverse transcription stage of viral replication [143]. A natural single-nucleotide polymorphism (SNP) was identified within the miR-28-3p target site in HTLV-1 subtype 1A isolates, corresponding to the Japanese strain ATK-1 [128]. Consequently, the ATK-1 virus sequence demonstrated less inhibition by miR-28-3p, suggesting that this miRNA may play a vital role in HTLV-1 transmission [128].

### 5.2. Tax-Specific miRNA

Numerous miRNAs have been identified that act synergistically with the HTLV-1 Tax protein and are transcribed through the NF-κB-mediated promoter transactivation. miR-146a was reported to be deregulated in HTLV-1-transformed cells and directly stimulated by Tax via NF-κB-mediated transactivation of the miR-146a promoter [146]. A single NF-κB site proximal to the transcription start point was responsible for this regulation [146]. Inhibition of miR-146a by anti-miRNA inhibitors suppressed the proliferation of HTLV-1-infected T cells, but not uninfected T cells, suggesting that miR-146a may serve as a potential therapeutic target for patients infected with HTLV-1 [161]. Tax similarly enhances expression of miR-155 in an NF-κB-dependent manner [147]. miR-155 was increased in HTLV-1-positive T-cell lines, promoting T-cell proliferation in these cells. miR-155 can also inhibit apoptosis by targeting the tumor-suppressor-protein-53-induced nuclear protein 1 (TP53INP1) [148]. miR-155 may act synergistically with miR-130b and miR-93—two miRNAs which also target TP53INP1 and demonstrate increased expression in HTLV-1-infected cells [149]. NF-κB has also been implicated in miR-34a regulation, with increased expression in HTLV-1-positive cell lines compared to human PBMCs and purified CD4+ T cells [150]. The ATLL-derived cell line, ATL-ED, does not express miR-34a and upon further investigation had methylation at the miR-34a promoter [150]. HTLV-1-infected cells that express miR-34a transcript contain binding motifs for NF-κB and p53, while expression of these factors appears to sustain miR-34a levels in infected cells [150]. The functional role of miR-34a in HTLV-1 infection includes regulation of target genes that influence cell turnover. Specifically, transfection of C91/PL cells with an miR-34a mimic downregulated mRNA targets SIRT1 and BAX, indicating that miR-34a inhibits apoptosis [150]. Certain miRNAs can also act as tumor suppressors and are downregulated in HTLV-1-infected cells. miR-31 negatively regulates the noncanonical NF-κB pathway by targeting NF-κB-inducing kinase (NIK), and is often silenced in ATLL cells through Polycomb-mediated epigenetic miRNA silencing [151]. Specifically, PBMCs derived from ATLL patients demonstrated significantly elevated methylation of histone H3K9 and H3K27 at the miR-31 locus, suggesting that aberrant suppressive histone methylation may contribute to the loss of miR-31 tumor suppression function in ATLL patients [151]. Tax can also mediate downregulation of certain miRNAs and has been described in association with chromatin remodeling. miR-149 and miR-873 play a role in regulating the histone acetyltransferases (HATs) p300/CREB-binding protein (p300/CBP) and p300/CBP-associated factor (PCAF), thereby controlling chromatin remodeling [152]. These chromatin modeling factors are known to play a critical role in HTLV-1 gene expression and are recruited by Tax to activate transcription [162,163,164,165]. Both miR-148 and miR-873 were downregulated in the HTLV-1-transformed cell line, MT-2, and when experimentally overexpressed in vitro, repressed CBP/p300, PCAF, and infectious viral particle release [165].

### 5.3. Hbz-Specific miRNA

The HTLV-1 viral protein Hbz is critical for disease development and has been associated with miRNA regulation. In addition to facilitating cell proliferation through Tax-mediated mechanisms, miR-155 and miR-146a have a functional role in ATLL cells, where Tax expression is often limited [146]. Specifically, miR-155 can target interferon regulatory factor 3 (IRF3) by antagonizing expression of inhibitor of nuclear factor kappa-B kinase subunit epsilon (IKKi) [71,163]. These immunomodulatory effects are potentiated by miR-146a, which targets and downregulates interleukin-1 receptor-associated kinases 1 and 2 (IRAK1, IRAK2) and tumor necrosis factor receptor-associated factor 6 (TRAF6) to ultimately weaken TLR and RLR signaling and block IFN and ISG expression [71,145,146]. While these immunomodulatory functions have not been specifically studied in the context of HTLV-1, it is possible that miR-155 and miR-146a may aid in the avoidance of immune detection, a critical feature of ATLL disease development [14]. Future studies should investigate potential interactions between these miRNAs and Hbz, given the key role of Hbz in ATLL pathogenesis.

The oncogenic potential of Hbz is exacerbated through Hbz-induced post-transcriptional activation of miR-17 and miR-21, which target the DNA-damage effector oligonucleotide/oligosaccharide-binding fold-containing protein 2A (OBFC2A) in CD4+ T cells [153]. OBFC2A encodes hSSB2, which disrupts ATM signaling and promotes activation of DNA repair and cell-cycle checkpoints [166,167]. Given the upregulation of miR-17 and miR-21 expression in HAM/TSP patients and correlation with Hbz expression, Hbz–miRNA-mediated downregulation of OBFC2A likely contributes to atypical cellular proliferation and genomic instability in disease states [153]. There is controversy as to whether miR-150-5p is upregulated or downregulated in HTLV-1-infected cells. miR-150-5p was initially found to be downregulated in HTLV-1-infected cells compared to healthy controls [154]. miR-150 downregulation was reported in HTLV-1-transformed cells and ATLL-derived cells, with consequential upregulation of STAT1 protein expression (required for continuous proliferation of HTLV-1-transformed cells) [155]. However, more recent studies have identified upregulation of miR-150-5p levels in HTLV-1 asymptomatic carriers compared to healthy controls and ATLL patients [156], which may be indicative of phasic expression of this miRNA in HTLV-1 cellular transformation and disease processes. Various miRNAs are downregulated in ATLL cells, which can be facilitated through Hbz-mediated impairment of Dicer [158]. The miRNAs miR-let-7a, miR-16, miR-20, miR-21, miR-31, miR-93, miR-125a, miR-132, miR-143, miR-155, miR-200, and miR-873 were identified as downregulated in acute ATLL patients through Hbz-mediated alteration in Dicer expression [158]. Hbz alters the expression of Dicer through removal of JunD from the AP-1 binding site in the Dicer proximal promoter, which can be rescued via the HDAC inhibitor valproate (VPA) [158]. Other studies have evaluated differential expression of miRNAs among healthy donors and ATLL patients using high-throughput analysis, identifying miR-let7a, miR-let7g, miR-181b, miR-26b, and miR-30c as differentially expressed miRNAs [159]. These miRNAs are directly associated with previously identified pathways in HTLV-1 pathogenesis, and primarily target genes involved in the MAPK pathway [159,168,169]. A recent review computationally analyzed 42 differently expressed miRNAs previously reported in healthy, HTLV-1-infected, and ATLL patients, identifying 12 miRNAs (miR-34a-5p, miR-146b-5p, miR-181b-5p, miR-26a-5p, miR-26b-5p, miR-222-3p, miR-155-5p, miR-193a-5p, miR-199a-3p, miR-199b-3p, miR-423-5p, and miR-150-5p) that likely impact biological pathways important in HTLV-1 pathogenesis [160]. Given the myriad roles miRNAs play in the HTLV-1 lifecycle and pathogenesis, future studies directed at therapeutic exploitation may be a viable chemotherapy alternative.

## 6. Transfer RNA (tRNA)

Transfer RNAs (tRNAs) range from 76 to 90 bases in length and are characterized by internal base pairing that gives rise to a characteristic stem-loop cloverleaf secondary structure [170]. tRNAs are essential for translation, acting as the vehicle that brings amino acids to the growing polypeptide chain [171,172,173,174,175]. Beyond translation, tRNAs possess several other functions. tRNAs can enzymatically catalyze the transfer of arginine from tRNA Arg to *N*-terminal Asp or Glu residues by Arg-tRNA-protein transferase, which facilitates protein degradation [176,177]. tRNAs can also respond to amino acid starvation by regulating the cellular proteome, whereby which tRNAs bind to the histidyl-tRNA synthetase (His-RS)-like domain in general control nonderepressible 1 (Gcn2) [178]. This in turn activates Gcn2 kinase activity, which will phosphorylate the eukaryotic translation initiation factor 2 (eIF2) and promote translation of general control nonderepressible 4 (GCN4) [178]. GCN4 encodes transcriptional activators that induce amino acid biosynthetic genes in response to amino acid starvation [179]. Specific to retroviruses, tRNA serves as the primer for reverse transcription to produce the dsDNA proviral genome [180]. HTLV-1 utilizes tRNA-Pro isoacceptors, in which the 3′ nucleotides act as primers that are perfectly complementary to the viral primer-binding site (PBS), a region within the 5′ UTR of the genomic RNA (gRNA) [10,181]. tRNA-Pro has recently been further characterized as a specific tRNA-Pro UGG isodecoder that contains a m1A58 modification to help facilitate plus-strand transfer during reverse transcription [181].

To express the gag–pol fusion protein, retroviruses often require ribosomal frameshifting to align different reading frames [182,183,184,185,186,187]. Frameshift signals are in part mediated by “shifty” tRNAs, aminoacyl-tRNAs located near the frameshift sites in HTLV-1 [186,187,188]. While the tRNAs used for this vary between retroviruses, Asn-tRNA has been identified as critical for translation within the ribosomal frameshift signal of HTLV-1 [189]. Interestingly, this tRNA often lacks the highly modified queuine (Q) base in its anticodon loop in infected cells, which correlates with tRNA involvement in frameshift events [189]. Codon usage can vary between retroviral and host translation, which can potentially be exploited therapeutically. Specific to HTLV-1, the codon CUA was determined by logistic computation to be differentially expressed between publicly available genomes for HTLV-1 and the non-disease-causing HTLV-2, which is responsible for encoding large hydrophobic residues (LHRs) that are essential in protein folding [190]. More recently, large sequencing studies have been employed to characterize differential tRNA expression profiles in the PBMCs of healthy control individuals, HTLV-1 asymptomatic carriers, and ATLL patients. Notably, 13 upregulated tRNAs and 17 downregulated tRNAs were identified in ATLL samples; however, the functional roles of these individual tRNAs have yet to be investigated [156].

tRNA fragments (tRFs) are ~19 nucleotide fragments produced from the 3′ ends of tRNA precursors of either end of mature tRNAs [191,192]. While tRFs in general are understudied, some are known to complex with Argonaute proteins or repress specific mRNAs through a microRNA-like mechanism [192,193]. Mass sequencing comparing differentially expressed small noncoding RNAs in normal T cells and HTLV-1-transformed T cells revealed that tRF-3019 was packaged into virions and capable of priming HTLV-1 reverse transcription [154]. This tRF corresponds to the 3′ end of tRNA-Pro, further support for its involvement in HTLV-1 reverse transcription [194].

## 7. *hbz* RNA

The existence of a viral transcript encoded by the antisense strand of the HTLV-1 provirus was first reported in 1989 [195]. Identified as *hbz*, this transcript is expressed from a TATA-less promoter in the 3′ LTR and is transactivated by SP1 [31,33,196,197,198]. *Hbz* is often the only viral transcript consistently expressed in ATLL cells [199]. This is partially attributed to the transcriptionally active 3′ region of the provirus (compared to hypermethylated 5′ LTR) protected by the presence of a viral CTCF binding site (insulator element) and a nearby enhancer element [33,200,201,202]. The *hbz* transcript and Hbz protein have distinct pathologic functions. Hbz can downregulate Tax-mediated sense transcription from the 5′ LTR promoter by competing with CBP/p300 [200]. Additionally, the Hbz protein can promote cell cycle progression and inhibit apoptosis through interaction with the retinoblastoma (Rb)/E2F-1 complex [203]. Importantly, Hbz protein can interact with the transcription factor JunD to promote expression of its own RNA [31]. This review will focus on the functional roles of *hbz* mRNA, which promotes proliferation of ATLL cells [33].

*Hbz* is a singly spliced transcript that is largely retained in the nucleus [204]. Studies that suppressed *Hbz* gene expression using siRNAs in ATLL-derived cell lines demonstrated decreased cellular proliferation. Various *Hbz* gene mutants were created that either altered RNA secondary structure or blocked protein synthesis to further dissect the role of *hbz* RNA and protein [33]. The proliferative effect in ATLL cells was only retained with an intact *hbz* transcript, and was not reliant on the presence of Hbz protein [33]. The *hbz* proliferative effect is partially mediated by *hbz* activation of E2F1 transcription and its target genes along with increasing G1/S cell cycle transition [33]. The fact that *hbz* is largely retained in the nucleus further supports that is has function beyond simply serving as a template for protein synthesis. More recently, the nuclear retention of *hbz* has been found to be independent of late events in HTLV-1 infection, such as cellular transformation [205,206]. Nuclear retention is largely due to the HTLV-1 3′ LTR promoter being inefficient at recruiting serine 2 phosphorylation (Ser2P) RNA polymerase II and polyadenylation factors, hindering nuclear export [206]. In conjunction with the Hbz protein, *hbz* mRNA enhances GATA3 transcription, promoting expression of CCR4 to facilitate T-cell expansion [207]. This function has been likened to lncRNA given the ability of *hbz* to associate with chromatin to regulate cellular proliferation [206].

The proliferative effect of *hbz* mRNA was further validated using an in vivo NOD/SCIDγchain−/− mouse model that forms solid tumors that infiltrate multiple tissues. Animals challenged with HTLV-1 leukemic T cells (SLB-1) with *Hbz* knockdown demonstrated significantly decreased tumor formation and organ infiltration compared to control groups, indicating that the proliferative role of *hbz* supports tumorigenesis [208]. While the Hbz protein promotes S-phase entry and apoptosis, the mRNA form attenuates apoptosis [209]. This is in part mediated by *hbz*-mediated enhancement of the promoter activity of survivin, an inhibitor of apoptosis, along with other cell-cycle-related genes [209]. The distinction between *hbz* mRNA and Hbz protein was further dissected through employment of mutant proviral clones that disrupted either RNA splicing, RNA secondary structure, or protein production of Hbz. Unlike the Hbz protein, which plays a critical role in in vivo viral persistence in New Zealand white (NZW) rabbits, specific point mutations that alter predicted *hbz* mRNA secondary structure, or loss of *hbz* mRNA, do not have a significant effect(s) on HTLV-1-mediated T-cell immortalization, in vivo persistence in NZW rabbits, or disease progression in humanized mice [210]. However, further studies are still required to characterize *hbz* mRNA interactions in T cells to dissect their role in viral pathogenesis.

## 8. Emerging RNA Technology

With increasing understanding of the diverse roles of RNA, improved sequencing methodology has rapidly emerged. Transcriptome sequencing permits deeper understanding of cellular gene expression and regulation, allowing investigators to uncover heterogeneity between normal and diseased states. However, traditional RNA-Seq can only detect average gene expression of a cell population and cannot parse out heterogeneity between individual cells [211,212]. The recent emergence of single-cell RNA sequencing (scRNA-Seq) now permits the study of genes and their molecular and functional alterations at the single-cell level. scRNA-Seq uses next-generation sequencing (NGS) to determine DNA and RNA expression patterns at the single-cell level. While slight variations exist among techniques, scRNA-Seq follows the same general workflow, beginning with single-cell isolation by micromanipulation, followed by laser-capture microdissection (LCM), microfluidics-activated cell sorting (FACS), cell lysis, reverse transcription of mRNA to cDNA, cDNA amplification, library preparation, and sequencing [213]. The most widely used technique for whole-genome amplification is multiple displacement amplification (MDA), which uses a Φ29 polymerase that minimizes chimeric sequence side products [214]. While this technique permits high genome coverage, sequence-dependent bias leading to over/under amplification in various genomic regions is a major caveat [213].

An alternative to scRNA-Seq is nanopore direct RNA sequencing (DRS), which allows for continuous reading of native RNA strands to provide a comprehensive database of individual RNA strands in cells. This technique is regulated by a helicase motor that controls RNA movement through a protein nanopore [215]. An applied voltage drives RNA through the pore, resulting in a monovalent ionic current that varies based on the nucleotide identity [215]. The specific ionic current signature can be translated into an RNA nucleotide sequence for individual RNA strands, which includes all exons, untranslated regions (UTRs), nucleotide modifications, and end modifications (5′ capping and 3′ polyadenylation) [215]. Unlike scRNA-Seq, nanopore sequencing permits direct detection of post-transcriptional modifications, such as nucleotide alterations and polyadenylation. Additionally, nucleotide reads can span thousands of base pairs to provide splice-variant and haplotype phasing [216]. The major drawback of this technique, however, is a relatively high error rate, which can be partially abated by including heterozygous variants and rigorous false-discovery rates (FDR) during allele-specific expression analysis [216].

A third-generation sequencing method alternative to nanopore is Pacific Biosciences24F (PacBio), which utilizes zero-mode waveguide wells to image nucleic acid synthesis processes, also known as a “sequencing-by-synthesis” method [217]. Currently, this method is primarily used for DNA sequencing, but in principle could be applied to single-molecule direct RNA sequencing through substitution of DNA polymerase with reverse transcriptase [218]. Few studies currently exist using PacBio for RNA sequencing over nanopore; however, this technique has been used to document m^6^A modification from various biological samples [219].

## 9. Future Areas of RNA Exploration and Therapeutic Intervention

The HTLV-1 transcriptome has proven to function beyond simply as a template for protein synthesis, and instead provides numerous functional and regulatory mechanisms to advance viral pathobiology. RNA epitranscriptomic modifications pose a significantly understudied field for HTLV-1; while m^6^A modifications have recently been documented and measured in HTLV-1, numerous other RNA modifications exist that have yet to be elucidated [95]. These include, but are not limited to, pseudouridine (Ψ) [220], inosine [221], 5-methylcytosine (m^5^C) [222], N6-methylguanosine (m^7^G) [223], N1-methyladenosine (m^1^A) [224], and N4-acetylcytidine (ac^4^C) [225]. Given the recent emergence of nanopore sequencing technology, qualification of these RNA modifications has become more feasible.

Various RNA-based therapies have already been studied for HTLV-1. Given the increasing understanding of genetic factors that promote HTLV-1 pathogenesis, siRNA delivery can be implemented to downregulate oncogenic genes. Using a peptide (p5RHH)-based nucleic acid delivery platform, siRNA targeting both NF-κB p65 and p100 was locally delivered to ATLL tumors, resulting in reduced mRNA and protein expression. Consequently, this anti-NF-κB nanotherapy significantly inhibited tumor growth in Tax-driven tumor mouse models as well as sensitized tumors to conventional chemotherapeutic agents [226]. While most miRNAs contribute to HTLV-induced disease by targeting pathways that enhance gene expression, miR-28-3p was found to repress the expression of HTLV-1 in a reporter assay by interfering with reverse transcription [131]. While miRNA can be delivered therapeutically, more investigation is required to determine how this miRNA behaves in vivo. Recently, an HTLV-1 envelope mRNA vaccine was developed and tested in NZW rabbits, demonstrating immunogenicity and protective antibody development [227]. Although a preventative HTLV-1 vaccine has yet to be tested in human clinical trials, the mRNA vaccine platform highlights an untapped use of RNA therapeutics in HTLV-1 disease. Compared to previous lab-animal-tested HTLV-1 vaccine candidates, immunization with an mRNA vaccine provides several benefits, including improved safety through the delivery of a noninfectious agent and circumvention of anti-vector responses [227].

tRNAs and tRFs are extensively documented in HTLV-1, yet therapies targeting these forms of RNA are understudied. One therapeutic method aimed at targeting tRNAs is inhibition of aminoacyl-tRNA synthetases (ARSs), a family of enzymes that ligate amino acids to their corresponding tRNAs in protein synthesis [228]. Pathogenic and human ARSs possess differences in sequence, structure, and topology, making them a viable target for pathogen-specific drugs [228]. In the context of HTLV-1, specific targeting of the ARSs associated with tRNA-Pro or tRF-3019 may impede viral reverse transcription to aid in post-exposure prophylaxis.

Targeting RNA epitranscriptomic modifications should not be overlooked as a potential therapeutic strategy. Given the recently documented role of m^6^A in regulating HTLV-1 viral transcripts and infectious viral particle release, targeting of this modification may aid in reducing transmission and infectivity [95]. The use of a small molecule inhibitor (STM2457) that targets the m^6^A writer protein, METTL3, was shown to diminish infectious viral particle release and sense-strand-derived viral transcripts in vitro, indicative that it may be therapeutically efficacious early in viral infection [95]. However, additional investigation is warranted evaluating how STM2457 behaves in an in vivo tumor growth model. While not yet fully evaluated in an ATLL model, STM2457 has been indicated as a viable therapeutic strategy for acute myeloid leukemia (AML), demonstrating that treatment leads to impaired engraftment and prolonged survival in various AML mouse models [83].

The study of the roles of RNA in HTLV-1 biology has revealed key functions of various forms of RNA in viral pathogenesis and disease development. However, recent attention on RNA epitranscriptomic modifications and their contributions to other viral pathogeneses and various cancers presents an untapped source for future investigation of HTLV-1. Continued investigation and understanding of the dynamic roles of RNA will continue to advance the field towards a better understanding of HTLV-1 pathobiology and aid in development of future therapeutic intervention.

## Figures and Tables

**Table 1:** Known miRNAs and associated effects in HTLV-1 pathobiology.

miRNA	Function	Target	Outcome	Reference
miR-28-3p	Antiviral	Genomic *gag*/*pol*	Induces post-entry block at reverse transcription level to inhibit viral replication and infection	[143]
miR-146a	Proviral	TRAF6	Induced by Tax to promote T-cell proliferation	[146]
miR-155	Proviral	TP53INP1	Inhibits tumor suppressor proteins to downregulate apoptosis; induced by Tax to promote T-cell proliferation	[147,148]
miR-130b, miR-93	Proviral	TP53INP1	Acts synergistically with miR-155 to downregulate apoptosis	[149]
miR-34a	Proviral	SIRT1, BAX	Contains binding motifs for NF-κB and p53; regulates gene expression of target genes implicated in T-cell turnover and apoptosis	[150]
miR-31	Antiviral	NIK	Negatively regulates noncanonical NF-κ signaling; silenced in ATLL cells through histone methylation	[151]
miR-149, miR-873	Antiviral	P300/CBP, PCAF	Downregulates transcription by regulating Tax-recruited chromatin modeling factors	[152]
miR-17, miR-21	Proviral	OBFC2A-hSSB2	Activated by Hbz to inhibit DNA repair and cell cycle progression	[153]
miR-150-5p	Not reported	STAT1, MYB, NOTCH1, NOTCH3	Inhibits activation of transcription factor STAT1; putative phasic upregulation/downregulation in HTLV-1	[154,155,156,157]
miR-let-7a, miR-16, miR-20, miR-21, miR-31, miR-93 miR-125a, miR-132, miR-143, miR-155, miR-200, miR-873	Antiviral	Not reported	Downregulated in ATLL- through Hbz-mediated Dicer impairment	[158]
miR-let7g, miR-181b, miR-26b, miR-30c	Not reported	MAPK pathway	Differentially expressed between healthy and ATLL patients	[159]
miR-34a-5p, miR-146b-5p, miR-181b-5p, miR-26a-5p, miR-26b-5p, miR-222-3p, miR-155-5p, miR-193a-5p, miR-199a-3p, miR-199b-3p, miR-423-5p, miR-150-5p	Not reported	Not reported	Differentially expressed between uninfected and HTLV-1-infected cells using computer-based prediction models	[160]
